# Editorial: Optimization of Exercise Countermeasures for Human Space Flight—Lessons From Terrestrial Physiology and Operational Implementation

**DOI:** 10.3389/fphys.2019.01567

**Published:** 2020-01-10

**Authors:** Jonathan P. R. Scott, Tobias Weber, David A. Green

**Affiliations:** ^1^KBR GmbH, Cologne, Germany; ^2^Space Medicine Team, European Astronaut Centre, European Space Agency, Cologne, Germany; ^3^Centre of Human and Applied Physiological Sciences, King's College London, London, United Kingdom

**Keywords:** microgravity, exercise countermeasures, human space exploration, cardiorespiratory, musculoskeletal

As we approach the 20th anniversary of the International Space Station (ISS), countermeasure (CM) exercise for human spaceflight has evolved from rudimentary physical activity into the complex, multi-modal programme that occupies ~25% of each ISS working day. However, despite the long history of CM exercise, questions remain regarding both its efficacy and effectiveness, in particular with respect to the challenge of managing cardiorespiratory (CR) and musculoskeletal (MS) adaptation during future human exploration missions (Scott et al.). This Research Topic (RT) was a result of a Workshop convened in January 2018 at the European Space Agency's (ESA) European Astronaut Centre (EAC) in Cologne, Germany. In a series of invited reviews, 52 authors from 31 institutions have synthesized current terrestrial exercise physiology knowledge and considered how this might be employed to optimize future CM exercise.

Hurst et al. examined high-intensity interval training (HIT), which involves repeated bouts of intense exercise, interspersed with periods of rest or lower intensity active recovery. HIT can be performed with a range of exercise modalities, including those already available on ISS and the authors concluded that terrestrial data support its use as a time-efficient approach to improve aerobic fitness. Interestingly, recent data also suggests beneficial neuromuscular effects, such as increased muscle strength and power, and jump performance. As such, employment of HIT-type protocols may provide a time-efficient alternative to current exercise CM approaches without requiring new hardware.

Ralston et al. examined the effects of single-set and three-set resistance training on muscle strength changes for different body segments (upper and lower body) and joint types (single and multi-joint training). They concluded that, while three-sets are more effective, particularly in trained individuals, single-set programmes can also produce significant increases in muscular strength. As three-set training entails significantly greater training volume, single-set training may be a useful approach in the busy pre-flight period. For space exploration, where mission and life support system resources (e.g., food, water, oxygen) will be at a premium, this also suggests that single-set resistance exercise, whilst not optimal, might still be sufficient.

The performance of aerobic and resistance training is termed “concurrent” training, which may negatively impact training effectiveness. Jones et al. conclude that, if strength and aerobic exercise must be performed on the same day, strength training should be employed first, and ideally >4 h prior to aerobic training. While operational constraints will always exist and crew preferences must be considered to maximize adherence, this suggests that, wherever possible, CM resistance training should be performed first and ‘back-to-back' (*i.e*. one immediately or shortly after the other) sessions (with aerobic training first) avoided.

One solution to the issue of concurrent training could be to use only one mode of exercise. Steele et al. examined the concept that adaptations to exercise are affected by the intensity of effort independent of modality. They concluded that, where effort and duration are matched, aerobic, and resistance training may produce broadly comparable physiological responses/adaptations, at least in terms of aerobic fitness and muscle strength/size. Thus, with appropriate protocols, one, uni-modal device might be sufficient for CM exercise, which has obvious advantages for exercise hardware provision and working volume requirements (Scott et al.). If one had to choose a mode, the authors conclude that resistance would provide the best outcomes. Candidate resistance training devices could be further simplified if the maximum training loads required were reduced. For instance, as reviewed by Behringer and Willberg, blood-flow restriction training requires only ~30% of the one repetition maximum (1RM) to increase muscle mass and strength, although it presents challenges, particularly in terms of its safe implementation, which could potentially limit adoption in-flight.

Although the review of Steele et al. raises the possibility that a uni-modal CM program might be sufficient, their conclusions are based only on aerobic fitness and muscle strength/size data. However, CM exercise must also manage skeletal adaptation. Thus, Gruber et al. examined the effect of the stretch-shortening cycle (SSC), during which muscle activation generates high muscle forces via elastic energy storage and, critically, high skeletal strains, and strain rates. The authors show that plyometric-type exercises, such as jumping and hopping, and to a lesser extent whole-body vibration, activate the SSC and promote muscle and bone adaption. Furthermore, a summary of recent bed rest data provides evidence that CR and MS adaptation can be managed with only 3-min of jumping exercise per day using a horizontal sledge-jump device.

Although a plyometric-based CM exercise programme would require new hardware and engineering integration solutions (e.g., vibration isolation), it suggests that spaceflight adaption could potentially be managed with a single, uni-modal device *and* consuming significantly less time and life support resources compared with the current multi-modal ISS programme. Moreover, as discussed by Laurens et al., reducing the total energy expenditure associated with CM exercise may also serve to reduce the risk of a negative energy balance and its associated consequences. With an improved understanding of the time course of CR and MS adaptation to both microgravity (μG) and CM exercise, the efficiency of a CM exercise programme could be further optimized by the interspersion of fixed periods of abstinence. Winnard et al. provided an initial analysis of muscle outcome measures from bed rest Control groups, reporting that “moderate” effects are evident by 7–15 days, and “large” effects by 28–56 days of unloading.

In the absence of body weight in μG and thus the requirement for postural control, significant adaptation occurs in the spine and its surrounding structures including the lumbopelvic and spinal muscles. This is associated with lower-back pain and possibly an increased risk of intervertebral disc herniation. The current ISS CM exercise programme is not optimized to activate the core musculature and Hides et al. reviewed terrestrial strategies for restoring muscle size and function that could be implemented or adapted for use in μG.

All of the above assumes that CM exercise device(s) are constantly available. However, a comprehensive CM strategy must consider the possibility that devices are not available (due to failure), cannot be used (due to crew injury) or use limited (due to mission dynamics). As such, this RT also considered “complementary” CM strategies that could enhance the effects of, or reduce reliance on, exercise. In this context, Willis et al. examined the influence of hypoxia on responses to exercise training, while Maffiuletti et al. provided an overview of neuromuscular electrical stimulation (NMES) and some practical recommendations as an adjunct to exercise. In the case of the later, whilst NMES-based resistance training has potential, it may not be suitable for all muscles and the skeletal and cardiovascular effects are still largely unknown. Finally, evidence reviewed by Guillot and Debarnot suggests that motor imagery (MI) improves motor performance and learning in a similar manner to actual practice of the corresponding movement. As such, when muscle activation is not possible during a mission, such as following a MS injury, MI may provide a strategy to minimize motor performance decrements, although definition of optimal approaches is needed.

In summary, the constraints of future space missions present unique challenges that must be addressed if exercise is to remain at the heart of the CM programme. We hope that this RT will contribute to the identification of strategies which, together, will result in an effective, efficient, and comprehensive CM strategy suitable for implementation irrespective of specific mission scenarios. With so many factors to consider and limited opportunities to evaluate new approaches, this will require an international effort. This effort could, and should, be led by the recently formed International Crew Health & Performance Working Group (ICHP), with representatives from the National Aeronautics and Space Administration (NASA), the Roscosmos State Corporation for Space Activities (Roscosmos), and the European (ESA), Japanese (JAXA) and Canadian (CSA) Space Agencies, chartered to facilitate coordination of requirements, risks, and capability demonstration plans for the forthcoming ‘Gateway' and beyond.

## Author Contributions

All authors contributed to the first draft of the manuscript, manuscript revision, and read and approved the submitted version.

### Conflict of Interest

JS, TW, and DG are all employed by KBR GmbH, Cologne, Germany.

